# Effects of NGR1 on the Protective Efficacy and Functional Vision Profile of Retinal Photodamage

**DOI:** 10.7150/ijms.122723

**Published:** 2026-01-01

**Authors:** Chi-Huang Chang, Jyh-Cheng Liou, Yi-Hsien Hsieh, Shih-Liang Yang, Chi-Wu Chang, Shao-Yun Tsai, Jui-Yi Yu, Ling Lu, Ping-Hsun Wang, Bo-Yie Chen

**Affiliations:** 1 Department of Ophthalmology, Chung Shan Medical University Hospital, Taichung City 40201, Taiwan, ROC.; 2 Department of Optometry, Chung Shan Medical University, Taichung City 40201, Taiwan, ROC.; 3 Institute of Medicine, Chung Shan Medical University, Taichung City 40201, Taiwan, ROC.; 4 Department of Chinese Medicine, Taichung Hospital, Ministry of Health and Welfare, Taichung City 40343, Taiwan, ROC.

**Keywords:** Notoginsenoside R1, cone opsin, photoreceptor, visual acuity, visual contrast sensitivity function

## Abstract

Prolonged exposure to high-intensity light can harm macula vision, particularly affecting the function of Müller cells and cone photoreceptors. Panax notoginseng saponins (PNS) have valuable pharmacological effects on cerebrovascular, neurological, and microcirculatory health. Notoginsenoside R1 (NGR1), derived from PNS, shows potential for treating vascular or ischemia-reperfusion-related retinal issues; however, its impact on cone cells and the functional vision profile is not well understood. This study aimed to explore the effect and efficacy of NGR1 on retinal photodamage *in vivo* in mice. In a mouse model, high-intensity light causes significant photoreceptor damage, increases the production of pro-inflammatory factors, promotes Müller cell gliosis, and remarkably reduces the content of M- and S-opsin in cones, resulting in the abnormal and dysfunctional mislocalization of cone-opsin protein trafficking. Our data demonstrated that NGR1 orally administered improved ERG amplitude, visual acuity, and visual contrast sensitivity function compared to the vehicle group. It also preserved S- and M-cone density, mitigated abnormal trafficking of cone opsin protein, inhibited Müller cell gliosis, and reduced retinal inflammation. Therefore, NGR1 may serve as a valuable traditional complementary therapeutic or nutraceutical component for enhancing functional vision and supporting the health of Müller and cone cells in the macula.

## Introduction

Long-term exposure to high-intensity digital light poses a significant threat to retinal health and vision, particularly among frequent screen users in developed countries. Specialized Müller cells and cone photoreceptors are concentrated in the human central macula, and cone opsin proteins in photoreceptors are essential for visual processing 1, 2. These opsin proteins are photosensitive G-protein-coupled receptors, which encapsulate 11-cis-retinal and, upon photon absorption, convert to their all-trans configuration 3. This transformation triggers the release and transport of all-trans retinal to the peripheral Müller cells or the retinal pigment epithelium (RPE), where enzymes recycle the retinal to regenerate the 11-cis-retinal form 2, 4, 5. To rapidly restore the light sensitivity of the central macula, cone opsins must be recharged with 11-cis-retinal 2, 3, 5. Excessive light exposure to Müller cells and cone photoreceptors can cause oxidative damage and inflammation, disrupting the recharge of the opsin protein with 11-cis-retinal, potentially leading to vision decline. To safeguard against these risks, it is crucial to take proactive measures such as administering natural supplements to protect Müller cells and cone-driven vision in the central macula.

*Panax notoginseng* is a medicinal plant with pharmacological properties found in various parts of the plant, especially in its roots 6-10. *Panax notoginseng* saponins (PNS) are widely used in healthcare products and have demonstrated beneficial effects on cardiovascular, cerebrovascular, neurological, and microcirculatory health 6-12. In ethnopharmacology, PNS has been shown to be effective in treating ischemia-reperfusion injury, circulatory disorders, metabolic syndrome, and diabetic eye conditions 6, 7, 9, 11, 12. The major compounds in PNS, such as notoginsenoside R1 and ginsenosides (Rg1, Rb1, and Rd) exhibit antioxidant and anti-inflammatory effects 13, 14. Compounds such as NGR1 and ginsenosides are found in retinal tissue following PNS administration, revealing their therapeutic potential 15. Moreover, PNS exhibits neuroprotective properties by inhibiting endoplasmic reticulum stress and protecting retinal ganglion cells (RGCs) from damage in both *in vitro* and *in vivo* studies 15-17. In animal studies, the intraperitoneal administration of PNS protected RGCs from ischemic damage 17. Additionally, the intraperitoneal administration of PNS and ginsenosides (Rb1 and Rd) can decrease high-energy light-induced oxidative damage to photoreceptors 18. Conversely, oral PNS mitigates blood-retinal barrier (BRB) disruption and inhibits inflammation in diabetic retinas 15, 17. Notably, a unique phytoestrogen in the PNS, NGR1, has been shown to enhance neural function by ameliorating synaptic dysfunction, improving oxygen transport, inhibiting inflammation, and activating protective signaling pathways 9, 19-27. Despite the established benefits of PNS via intraperitoneal or oral administration 18, further investigation is needed on the effects of NGR1 on the protective efficacy and functional vision profile in retinal photodamage, particularly the cone function, which presents a potential breakthrough in central vision treatment.

The LED-induced retinal photodamage model helps to evaluate the effects of natural compounds on the functionality of photoreceptors 28-32. Light-induced damage to retinal photoreceptors and stress on Müller cells impair retinal function 18, 28, 31. Previous research has shown that exposure to LED light causes cone dysfunction and disorders in cone-opsin protein trafficking, resulting in reduced functional vision in mice 28, 31, 33. This study aimed to examine the protective effects of NGR1 on functional vision profile and to clarify whether NGR1 plays a role in reducing Müller cell abnormalities and decreasing its pro-inflammatory response, thereby protecting cone opsin trafficking adequately and preserving cone photoreceptor function.

## Materials and Methods

### Animals

This study was approved by the Institutional Animal Care and Use Committee of Chung Shan Medical University (IACUC 113134). Female CD1® (ICR) albino mice (n = 30, 8-10 weeks old, 25-28 g) were obtained from BioLASCO Taiwan Co., Ltd. The mice were housed in a room maintained at a standard temperature (21~24 °C), humidity (45~70%) and luminance (12/12 hour cycle from 07:00 to 19:00, with roughly 100-300 lux during the light phase and 15-30 lux during the dark phase under standard conditions) during the experiments.

### Experimental design and animal grouping

**Figure 1:**
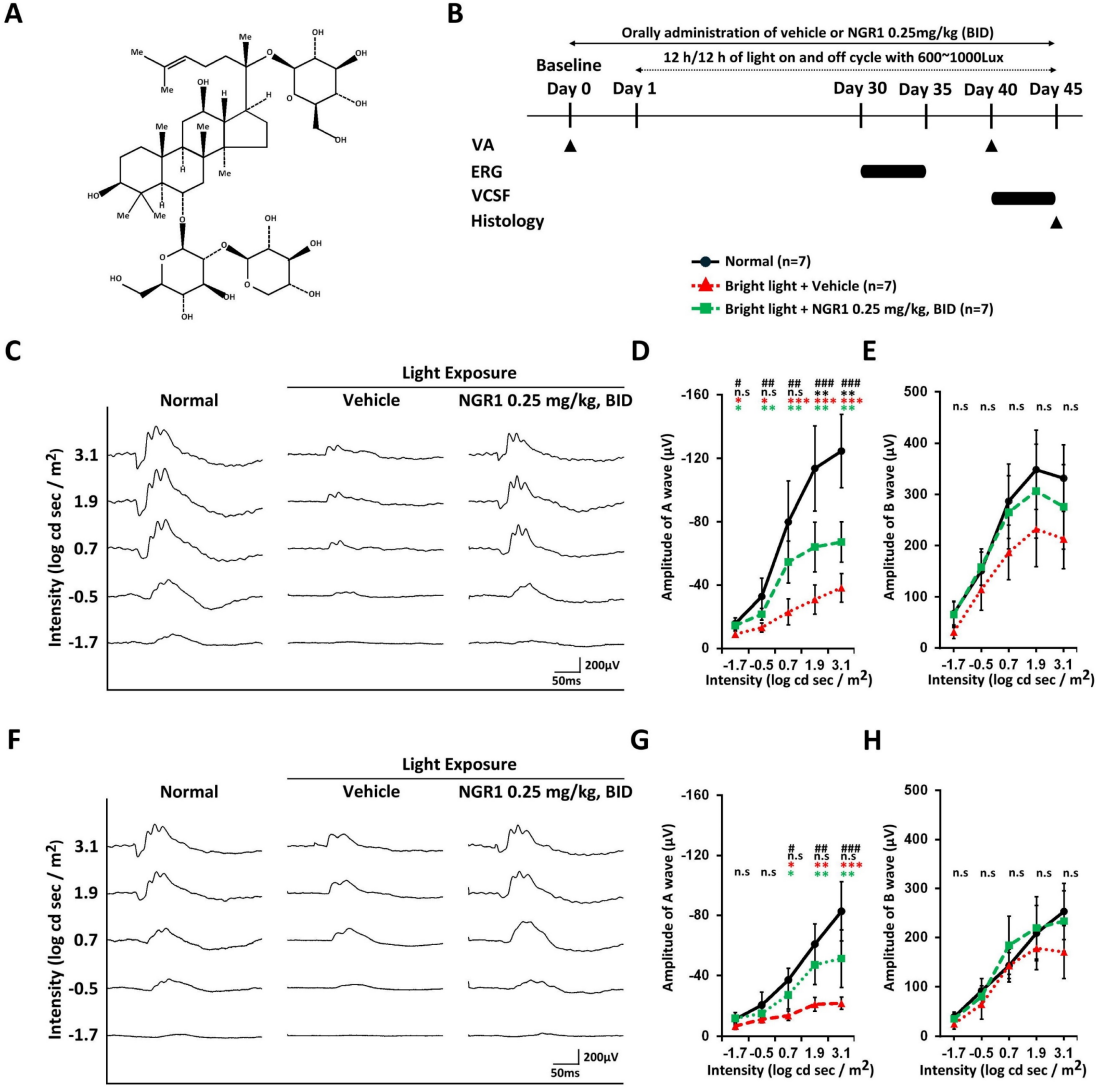
Protective effect of NGR1 on light-evoked photoreceptor function deterioration. **(A)** Chemical structure of notoginsenoside R1 (NGR1). **(B)** Timeline of the experimental design. **(C)** Representative ERG waveforms elicited by green light stimulation under dark-adapted conditions. **(D, E)** Average of a and b waves elicited by green light stimulation under dark-adapted conditions. **(F)** Representative ERG waveforms elicited by ultraviolet (UV) light stimulation under dark-adapted conditions. **(G, H)** Average amplitude of a and b waves elicited by UV light stimulation under dark-adapted conditions. Group differences were assessed by Kruskal-Wallis test, if significant (#p < 0.05, ##p < 0.01, and ### p < 0.001); pairwise comparisons were performed using the Mann-Whitney U test. * p < 0.05, **p < 0.01 and *** p < 0.001 indicated the significance. Black asterisks denote Normal vs. Light exposure + NGR1 0.25 mg/kg group; green asterisks denote Light exposure + NGR1 025 mg/kg vs. Light exposure + Vehicle, and red asterisk denote Normal vs. Light exposure + Vehicle.

NGR 1 (ChemFaces, China) (Figure 1A) was dissolved in a vehicle (H_2_O with 10% propylene glycol 400) and administered orally to a mouse, the minimum dosing interval was 10 hours, with administration typically at 07:00 to 08:30 and 18:30 to 20:00 each day. The model of retinal photodamage was induced by LED light (600-1000 lux, 12-h light-on/off cycle daily from Day 1 to Day 45) 31. Vehicle or NGR1 supplements were administered orally at baseline (1 day before light exposure) and throughout the experiment. Visual acuity (VA) was assessed at baseline (before light exposure) and on day 40. Visual contrast sensitivity function (VCSF) was assessed from day 40 to day 45, and electroretinography (ERG) was performed from day 30 to day 35 (Figure 1B). First, the effective oral doses of NGR1 were evaluated in a pilot study using VA analysis. Mice were administrated NGR1 at 0.025 mg/kg twice daily (BID; n = 3) and 0.25 mg/kg BID (n = 3). The doses were chosen based on preliminary VA analysis to identify effective treatment in this mouse model (Supplementary table 1). Second, the protective efficacy of NGR1 against functional vision loss was determined using ERG, VA, VCSF, and retinal tissue analysis. The remaining mice were randomly divided into three experimental groups: (1) normal (no light exposure; n = 8); (2) light exposure + vehicle (BID) group (n = 8); and (3) light exposure + NGR1 (0.25 mg/kg, BID) group (n = 8). The mice were maintained under complete darkness conditions overnight before being assessed for ERG, VA, and VCSF or sacrificed for histological analysis. The experiments were conducted in three independent cycles, and data were analyzed in a single-blind method to serve as the basis for subsequent statistical analyses.

### *In vivo* ERG analysis

The mice were dark-adapted overnight and anesthetized intraperitoneally with thiopental (50 mg/kg). Pupil dilation was achieved using eye drops (1% atropine, 2.5% phenylephrine, and 0.5% proparacaine), and the corneas were lubricated with 2.5% hypromellose gel. The mice were placed on a heating pad during the experiment. Electrodes were applied to the corneas, with a ground electrode on the tail and a reference electrode on the scalp. A Phoenix MICRON™ Ganzfeld ERG System recorded ERG a-wave and b-wave responses; the right eye was calibrated with green flashes (-1.7 to 3.1 log cs s/m^2^), and the left eye was calibrated with UV light flashes (-1.7 to 3.1 log cs s/m^2^).

### *In vivo* VA and VCSF analysis

Functional vision analyses of VA and VCSF in the mice were performed using the optomotor response method 28, 31. The setup involved striped grating patterns at six spatial frequencies (0.033, 0.055, 0.082, 0.164, 0.328, and 0.437 cycles per degree (cpd) and ten contrast levels (10-100%) 28, 31. Individual thresholds were determined, and an inverted U-shaped VCSF curve was plotted to assess visual performance 28, 31. The area under the curve represents the VCSF visibility index, with faster responses to lower-contrast stimuli indicating higher visibility and better functional vision.

### Histological and immunohistochemistry (IHC) analyses

Mice were sacrificed via CO_2_ inhalation on day 46. The right eye from each mouse was enucleated, fixed for 24 h, and embedded in paraffin. Sagittal retinal sections (5 µm) were stained with hematoxylin and eosin and underwent IHC. Analyses were performed using an Olympus CX-22 microscope at 200× magnification. The thickness of the outer nuclear layer (ONL) and outer segment-inner segment (OS-IS) regions was measured 1.0 mm superior and inferior (n = 8 per group). Antigen retrieval was performed using sodium citrate buffer, followed by incubation with M opsin (1/500, Cat. No. NB110-74730; Novus Biologicals, Littleton, CO, USA); S-opsin (1/500, Cat. No. NBP1-20194, Novus Biologicals, Littleton, CO, USA), rhodopsin (1/500, Cat. No. ab98887; Abcam, Cambridge, UK) and glial fibrillary acidic protein (GFAP) antibody (1/400, Cat. No. ab7260, Abcam, Cambridge, UK). The average number of labeled cells in the ONL, located 1.0 mm superior and inferior, was analyzed (n = 6 per group). M opsin proteins are typically found in the OS of the normal retina. Mislocalization of M opsin in the ONL indicates photoreceptor dysfunction.

### Protein analysis

The left -eye retina of the mouse retina was isolated and homogenized in 50 μL of lysis buffer (25 mM Tris-HCl, pH 7.5; 100 mM NaCl; 1% Nonidet P-40) and diluted up to a 5 mg/mL protein concentration for quantifying proinflammatory factors using a multiplex bead array assay (Bio-Plex Pro™ Mouse Cytokine Assay, Bio-Rad Laboratories). The average concentrations of proinflammatory factors were assessed (n = 5 per group), and the changes in the levels of interleukin 1 beta (IL-1β), interleukin 12 _p70_ (IL-12 _p70_), interleukin 13 (IL-13), monocyte chemoattractant protein 1 (MCP-1), macrophage inflammatory protein 1 beta (MIP-1β), and tumor necrosis factor-alpha (TNF-α) were analyzed.

### Statistical analysis

Statistical data are presented as the mean ± standard error and were analyzed using the Statistical Package for the Social Sciences software (IBM Corp., Armonk, NY, USA). Differences between the experimental groups were assessed using the Kruskal-Wallis and Mann-Whitney U tests, with p values < 0.05, 0.01, and 0.001 indicating statistical significance. The hash symbol (#) in the figure denotes the significance of nonparametric Kruskal-Wallis test used to assess distribution differences among multiple groups. If a difference is detected, pairwise comparisons between groups were conducted using the Mann-Whitney U test. An asterisk (*) indicates statistical significance.

## Results

### Orally administered NGR1 preserves the photosensitive function of photoreceptors

In the first instance, we coordinated a pilot study to evaluate the effectiveness of the oral dose of NGR1 under light exposure and established 0.25 mg/kg BID as the effective dose based on VA outcomes in the mouse model of retinal photodamage (Supplementary Table 1). On day 40, VA performance was 0.273 ± 0.073 cycles per degree (cpd) at 0.25 mg/kg BID, compared with 0.109 ± 0.036 cpd at 0.025 mg/kg BID and 0.103 ± 0.031 cpd in the vehicle group (Supplementary Table 1). We examined the effects of NGR1 (0.25 mg/kg, BID) on retinal function in light-exposed mice using ERG assessments at 30-35 days post-exposure (Figure 1B). Dark-adapted, green light-evoked ERG analysis revealed a mixed response from the rods and M-cones (Figure 1C). The a- and b-wave amplitudes (Figure 1C, D, E) were reduced in vehicle-treated mice compared to normal mice, indicating retinal dysfunction due to high-energy light stress. Orally administered NGR1 attenuated the loss of a- wave amplitude and showed a partial reduction in the declining trend of b-waves amplitude (Figure 1C, E, and E). In dark-adapted UV light-evoked ERG analysis (Figure 1F, G, H), vehicle-treated mice showed reduced a-wave amplitude and partial b-wave amplitudes, may indicating a decline in the S-cone response. In contrast, the oral administration of NGR1 alleviated the reduction in the amplitudes of the a-wave and partial b-waves response (Figure 1F, G, and H). No significant differences in implicit times were observed between the groups (Supplementary Figure 1A, B, C, and D). These results indicate that NGR1 treatment (0.25 mg/kg, BID) may preserves the photosensitive function of photoreceptors in high-energy light-exposed mice.

### Orally administered NGR1 preserves functional vision against light-induced deterioration

**Figure 2:**
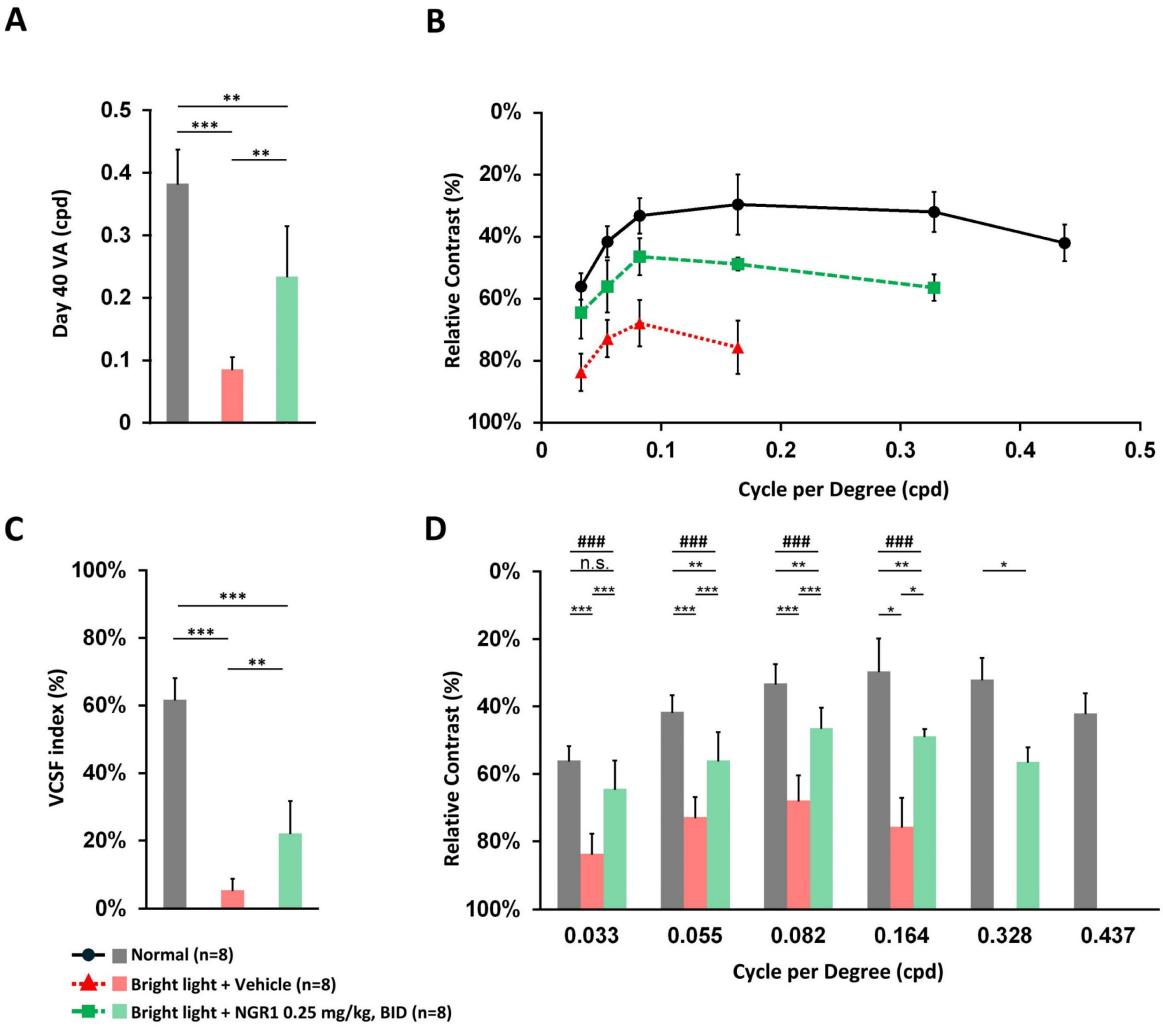
Protective effect of NGR1 after 40 days of light exposure-evoked VA and VCSF deterioration. **(A)** VA threshold on day 40. **(B)** Threshold in the reversed U-shaped VCSF curve on days 40-45. **(C)** Comparison of the VCSF index of each group. **(D)** Comparison of individual contrast function in different cycles per degree (cpd). Group differences were assessed by Kruskal-Wallis test, if significant (#p < 0.05, ##p < 0.01, and ### p < 0.001); pairwise comparisons were performed using the Mann-Whitney U test, * p < 0.05, **p < 0.01, and *** p < 0.001 indicated the significance.

VA deterioration was observed 40 days after light exposure in the vehicle group (Figure 2A). In contrast, the oral administration of NGR1 significantly attenuated VA loss. The NGR1-treated mice had a higher VA threshold (0.234 ± 0.080 cpd) than that of the vehicle group (0.086 ± 0.020 cpd) (p < 0.01; Figure 2A). On days 40 to 45, the vehicle group showed a trend of decline in the VCSF curve after light exposure, producing a smaller curve than the unexposed normal group (Figure 2B). In contrast, the NGR1 group exhibited better VCSF curves and preserved high spatial-frequency visual performance (Figure 2B). As a result, the VCSF visibility index, which represents the area under the VCSF curve, was significantly higher in the NGR1 group (22.12 ± 9.62%) than in the vehicle group (5.37 ± 3.32%; p < 0.001) (Figure 2C). Notably, the NGR1-treated mice exhibited preserved performance in high spatial frequency VCSF during the 0.328 cpd analysis (56.41 ± 4.27%) (Figure 2D), which was not detectable in the vehicle group, that indicated that NGR1 partially preserved visual performance relative to the vehicle group. Additionally, NGR1-treated mice showed better thresholds at middle-to-low spatial frequencies in the VCSF than in the vehicle-treated group (p < 0.01; Figure 2D). The results for the NGR1 treated group were as follows: 0.164 cpd at 48.8 ± 2.1%, 0.082 cpd at 48.9 ± 7.6%, 0.055 cpd at 55.3 ± 8.1%, and 0.033 cpd at 62.8 ± 8.5%. In contrast, the results of the vehicle group were: 0.164 cpd at 75.6 ± 8.5%, 0.082 cpd at 67.9 ± 7.5%, 0.055 cpd at 72.4 ± 5.7%, and 0.033 cpd at 82.1 ± 7.1% (Figure 2D). These results indicated the efficacy of NGR1 *in vivo*, potentially improving functional vision against light-induced deterioration.

### Orally administered NGR1 preserves retinal and photoreceptor integrity against light-induced retinal damage

**Figure 3:**
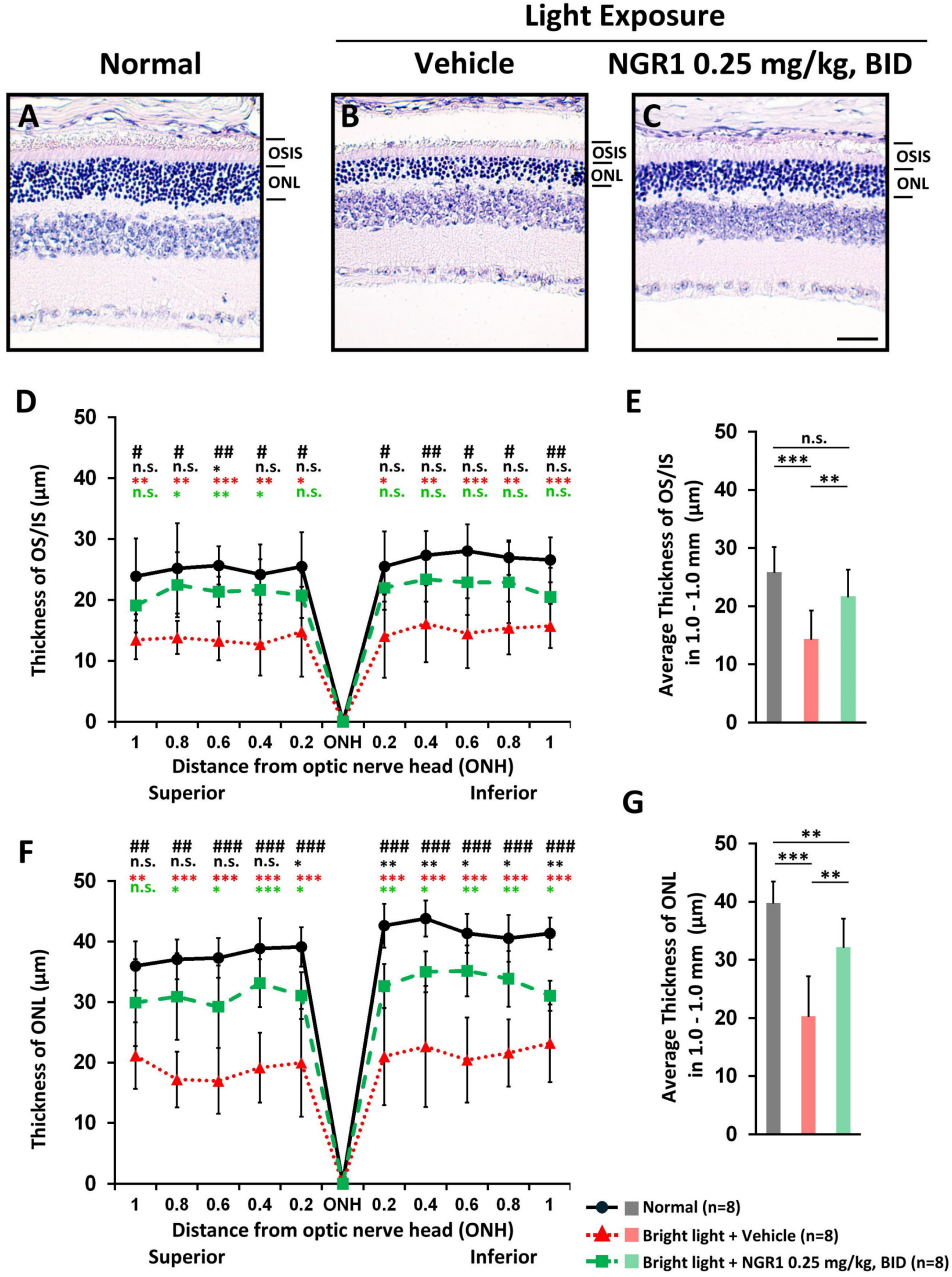
Protective effect of NGR1 after 40 days of light exposure-evoked photoreceptor degeneration. Hematoxylin and eosin (H&E) staining of **(A)** normal, **(B)** light exposure + vehicle, and **(C)** light exposure + NGR1 0.025 mg/kg groups. **(D)** Residual thickness of the OS/IS layer within 1.0 mm superior and inferior to the optic nerve head. **(E)** Average thickness of the OS/IS layer. **(F)** Residual thickness of the ONL layer within 1.0 mm superior and inferior to the optic nerve head. **(G)** Average ONL thickness. Group differences were assessed by Kruskal-Wallis test, if significant (#p < 0.05, ##p < 0.01, and ### p < 0.001); pairwise comparisons were performed using the Mann-Whitney U test, * p < 0.05, **p < 0.01, and *** p < 0.001 indicated the significance. Black asterisks denote Normal vs. Light exposure + NGR1 0.25 mg/kg group; green asterisks denote Light exposure + NGR1 025 mg/kg vs. Light exposure + Vehicle, and red asterisk denote Normal vs. Light exposure + Vehicle. Scale bar: 40 µm.

On day 45, a vertical histopathological examination of the retinas and analyses of OS-IS and ONL integrity were performed. Compared with the unexposed normal group (Figure 3A), the photoreceptor layer was notably affected in the vehicle group after light exposure, with a marked decrease in both ONL and OS-IS thicknesses (Figure 3B). The NGR1-treated mice preserved the thickness of the OS-IS (p < 0.001, Figure 3C, D, E) and ONL (p < 0.001, Figure 3C, F, G) significantly better than the vehicle group.

**Figure 4:**
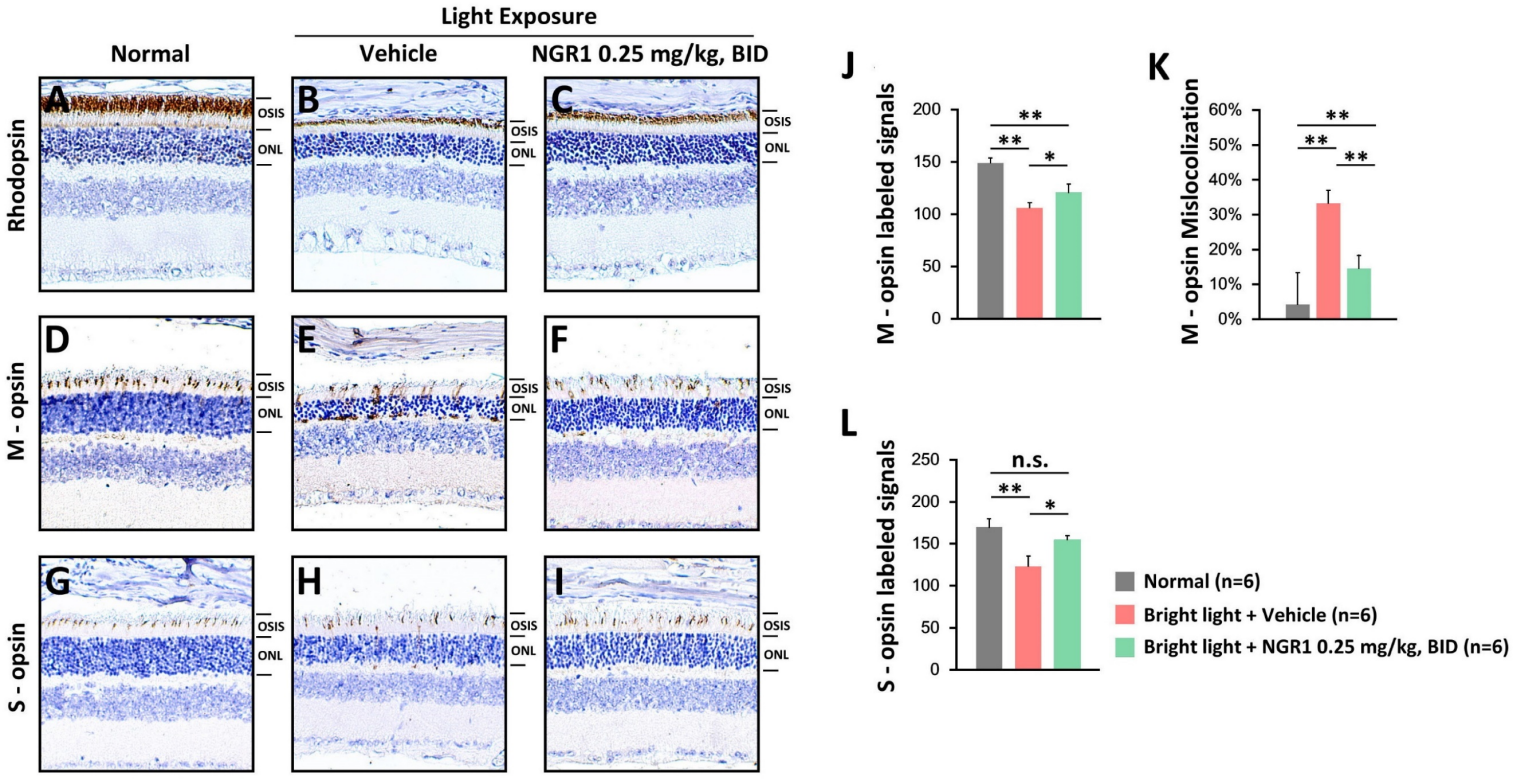
Protective effect of NGR1 on photoreceptor function. Expression of rhodopsin protein **(A-C)**, cone M opsin protein **(D-F)**, and cone S opsin protein **(G-I)** in the normal, LED + vehicle, and LED + NGR1 0.025 mg/kg groups. **(J)** M opsin-labeled cell density. **(K)** Percentage of M opsin mislocalization. **(L)** S opsin-labelled cell density. Pairwise comparisons were performed using the Mann-Whitney U test; * p < 0.05, **p < 0.01, and *** p < 0.001 indicate significance. Scale bar: 40 µm.

Rod and cone photoreceptors were assessed using IHC. The unexposed control group displayed rhodopsin-labeled, thick OS layers (Figure 4A). After light exposure, the vehicle group exhibited weakened rhodopsin-labeling in the OS layers (Figure 4B), whereas the NGR1 group maintained robust rhodopsin-labeled OS layers (Figure 4C). Furthermore, the vehicle group experienced a decline in M- and S-opsin-labeled cone cell densities (Figures 4 E, H), whereas the NGR1-treated group preserved these crucial densities (Figures 4F and I). There were significant differences in the M- and S-opsin-labeled cone cell densities between the vehicle- and NGR1-treated groups (Figures 4J, L). Notably, the vehicle group exhibited significant abnormalities in the trafficking and localization of M opsin within the ONL (Figure 4E, K). However, NGR1 treatment effectively prevented this effect (p < 0.001; Figure 4F, K). These findings indicate that NGR1 treatment effectively protects cone density and modulates retinal function following light exposure, suggesting significant implications for functional vision.

### NGR1 attenuated retinal inflammation caused by high-energy light exposure

**Figure 5:**
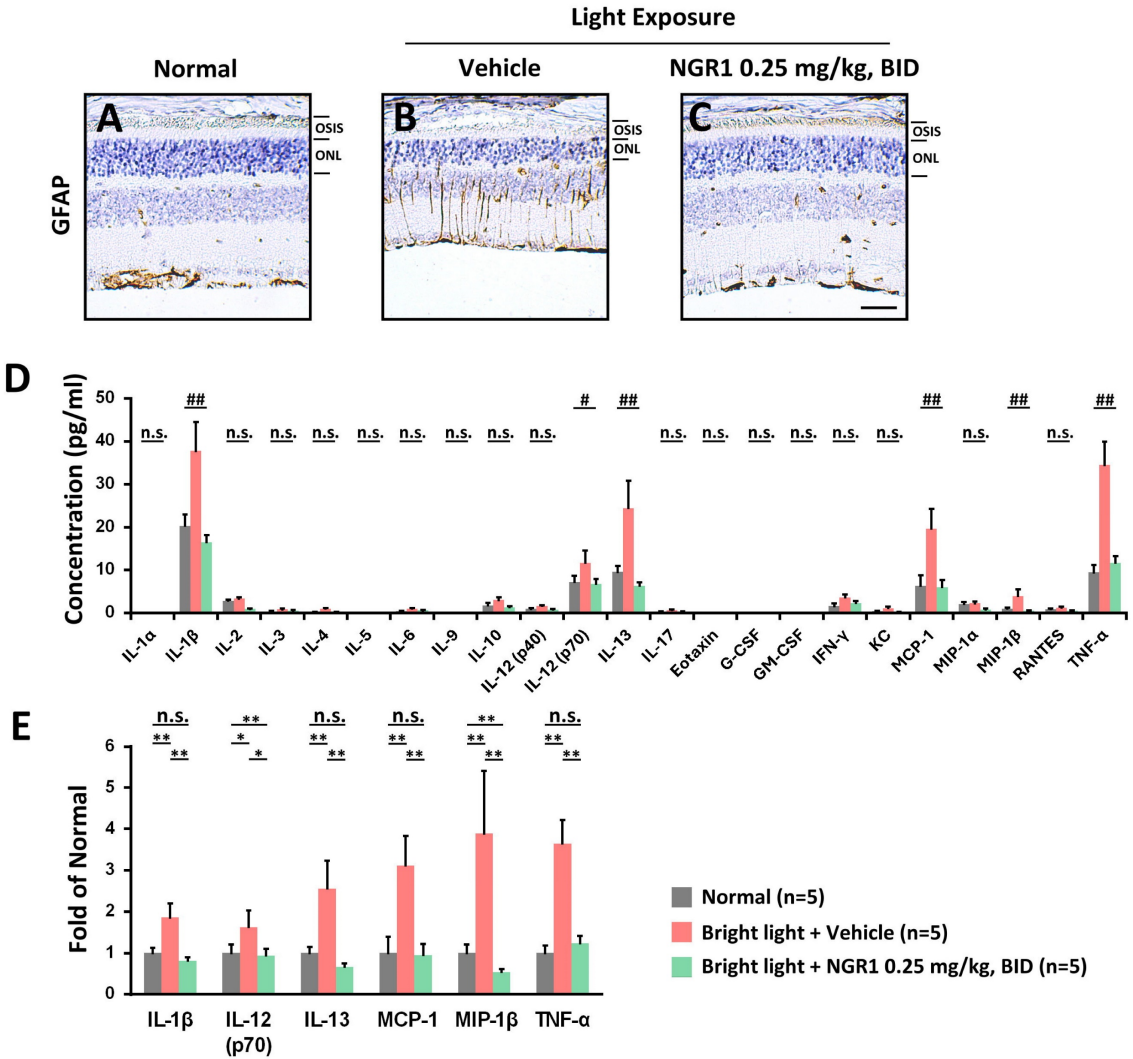
Protective effect of NGR1 on the risk of retinal inflammation. The expression of glial fibrillary acidic protein (GFAP)-labeled signals in **(A)** normal, **(B)** LED + vehicle and **(C)** LED + NGR1 0.025 mg/kg groups. **(D)** Concentrations of inflammatory risk factors in the retina. **(E)** Fold-change in the expression of inflammatory risk factors in the retina. Group differences were assessed by Kruskal-Wallis test, if significant (#p < 0.05, ##p < 0.01, and ### p < 0.001); pairwise comparisons were performed using the Mann-Whitney U test, * p < 0.05, **p < 0.01, and *** p < 0.001 indicated the significance. Scale bar: 40 µm.

Chronic exposure to high-energy light can severely damage retinal health, causing oxidative stress and inflammation, which disrupts synaptic activity and impairs the functions of Müller cells and photoreceptors. It is known that Müller cells express the glial fibrillary acidic protein (GFAP) in response to retinal oxidative damage 34. Compared with the age-matched normal retina (Figure 5A), the retina of the vehicle group exhibited damage and dysfunctional gliosis of Müller cells (Figure 5B), which was accompanied by increased GFAP protein expression following light exposure. However, the administration of NGR1 was able to reduce the light-induced damage and stress experienced by Müller cells (Figure 5C). We further evaluated the inflammatory retinal profile induced by light exposure and the response to NGR1 administration (Figure 5D). The vehicle group after light exposure demonstrated a significant increase in the content of interleukins (IL-1β, IL-12_p70_, IL-13), MCP-1, MIP-1β, and TNF-α. However, these levels were significantly lower in the NGR1 group (p < 0.01) (Figure 5E). These findings strongly suggest that oral administration of NGR1 not only protects Müller cells and photoreceptor function but also effectively reduces retinal inflammation, offering a promising approach for preserving vision during increased light exposure.

## Discussion

Specialized photoreceptors are crucial for photosensitivity and vision initiation. Damage to the integrity of the neural retina, particularly the degeneration of Müller cells and cones, can lead to severe conditions such as age-related macular degeneration and diabetic retinopathy, which significantly affect vision 15, 35, 36. In the present study, we investigated the protective efficacy and underlying cellular effects of NGR1 in a mouse model of high-energy light-induced photoreceptor dysfunction. Our findings demonstrate that a low oral dose (0.25 mg/kg, BID) significantly preserves functional vision by maintaining the density of M- and S-cones, inhibiting mistrafficking of M opsin, and alleviating inflammatory stress in the retinal tissue after retinal photodamage. Additionally, NGR1 helps protect Müller cell integrity and inhibits high-energy light-induced abnormal hyperplasia while protecting inner BRB functionality.

The central macula in humans is essential for high-resolution vision and is particularly sensitive to high spatial frequencies 37, 38. Declines in VA and VCSF can significantly affect quality of life and task performance 39-41. High spatial frequency is crucial for achieving optimal functional vision, and the evaluation of VA and VCSF, in addition to ERG, is vital for understanding the effects of natural ingredients on retinal health 15, 28, 31, 42. In the ERG analysis, we measured the amplitude responses to dark-adapted green light-evoked stimuli, which reflected a mixed rod-cone (M opsin) function. Dark-adapted UV light responses indicate S-cone function 43-45. Our ERG tracing study highlighted that NGR1 effectively preserved the amplitude of retinal a- and b-waves in response to both green and UV light despite high-energy light exposure (Figure 1). Furthermore, we revealed that NGR1 intervention significantly mitigated the detrimental effects of high-energy light exposure on high spatial frequency visual performance, as demonstrated by improvements in VA and VCSF thresholds in mice (Figure 2). In particular, aligned with the results of the VA, VCSF, and ERG analyses, the cellular analysis of retinal tissue indicated that NGR1 contributes to maintaining M- and S-cone density, preserving photosensitive function and ensuring proper trafficking of M opsin in retinal tissue against light-induced damage (Figure 4). Collectively, these results strongly underscore the pharmacological benefits of NGR1 on cone photoreceptors in addition to rods and the overall protection of vision functionality, suggesting that it is a candidate for enhancing visual health.

Following the administration of PNS, crucial components, such as ginsenosides (Rg1, Rb1, and Rd) and NGR1, were distinctly identified in the retinal tissue 15. While orally administered NGR1 may reach the retina, its transit involves gastric stability, absorption mechanisms, and BRB crossing, each requiring clarification to accurately interpret retinal exposure and effects; thus, comprehensive pharmacokinetic and BRB-penetration studies are warranted. Although these components individually demonstrate similar bioactivities, their interactions with herbal formulas can yield diverse or even contradictory effects owing to variations in the ingredients, glycosyl linkages, bioavailability, and other critical factors 6, 17, 46, 47. The relatively low prevalence of unique saponins in the PNS complicates the study of the specific pharmacological benefits of these active components, shifting the focus towards the overall effects of total saponins. Most pharmacological investigations of these individual components have been confined to *in vitro* studies, leaving a significant gap for *in vivo* research and limiting functional exploration. However, another study reported that intraperitoneal pretreatment with PNS (50 mg/kg and 200 mg/kg) may involve combinations of active components that could ameliorate light-induced retinal degeneration and mitigate the loss of the ERG response 18. In our current study, we demonstrated that the single component, NGR1 can be effectively administered orally at a low dosage (0.25 mg/kg, BID) to protect photoreceptor function. Our rigorous analyses of the functional vision profile of VA, VCSF, ERG, and cellular examination of the retinal tissue provide strong evidence supporting this effect.

A recent study demonstrated that NGR1 administered orally at a dose of 30 mg/kg exhibited antioxidant and anti-inflammatory effects 48. It significantly reduced the production of harmful reactive oxygen species in high-glucose-treated rat retinal Müller cells and diabetic-obese mice 48. Furthermore, NGR1 was also shown to mitigate oxidative damage in retinal endothelial cells by modulating redox activity 49. Our findings revealed that retinal photodamage induces the abnormal activation of Müller cells and elevates levels of inflammatory factors, including IL-1β, IL-12p70, IL-13, MCP-1, MIP-1β, and TNF-α (Figure 5). However, the source of these inflammatory factors may be damaged RPE, hyperplasia of Müller glia, or infiltrating immune cells that pass through the compromised inner BRB. In retinopathic conditions, the excessive production of MIP-1β and MCP-1 contributes to vascular injury 50, while cytokines such as TNF-α and IL-1β lead to various neurodegenerative effects on retinal cells 51. Additionally, IL-1β, TNF-α, and MCP-1 play crucial roles in leukocyte infiltration, inflammation, and the dysfunction of the BRB 52. Notably, we found that oral treatment with NGR1, even at a relatively low dosage (0.25 mg/kg, BID), significantly mitigated stress on Müller cells, adjusted retinal inflammation, and particularly modulated M opsin traffic and function in cone cells. However, protecting the normal status of Müller glia may support the maintenance of the cone-specific retinoid cycle, thus providing regenerated 11-cis-retinal to reactivate opsin proteins and aiding proper opsin trafficking to the outer segments of cones, in addition to preserving the function of the inner BRB. Although albino CD1 (ICR) mice exhibit certain physiological differences from humans, the present findings have biopharmacological relevance. Nevertheless, the translational applicability of these results to clinical settings requires further testing and evaluation. In the present study, this evidence strongly supports the potential of NGR1 as a therapeutic or nutraceutical agent to enhance central macular function.

Emerging nutraceutical and pharmacological therapies show great promise for slowing photoreceptor decline; however, many struggle to restore vision under clinical retinal conditions. It is important to note that our experiments used ICR mice, and species-specific differences may limit direct extrapolation to human clinical settings. In the other hands, compounds such as crocin, crocetin, lutein, zeaxanthin, and mesozeaxanthin are known for their visual benefits 31, 53-56; however, there is an urgent need for new solutions to enhance cone-rich macular function against high-intensity digital light.

## Conclusion

The present study revealed that NGR1 may uniquely target the M opsin protein, correct M opsin trafficking, prevent high-energy light-evoked abnormalities in cone photoreceptor cells, and protect Müller cells for maintaining a cone-specific retinoid cycle supply, thereby improving their functionality. This component could potentially restore cone cell function and enhance synaptic plasticity before the onset of degeneration. However, further investigation is required.

## Supplementary Material

Supplementary table and figure 1 which depicts the average implicit times of the a- and b-waves elicited by green and UV light stimulation under dark-adapted conditions.
