# Supplementary material for: Effects of NGR1 on the Protective Efficacy and Functional Vision Profile of Retinal Photodamage
Source: Int J Med Sci. 2026 Jan 1;23(2):378–88. doi: 10.7150/ijms.122723 (PMC12825012; doi:10.7150/ijms.122723)
Supplement: Supplementary file 1 — Supplementary table and figure 1 which depicts the average implicit times of the a- and b-waves elicited by green and UV light stimulation under dark-adapted conditions. [file ijmsv23p0378s1.pdf]

Pilot Study Groups	VA at Baseline (cycle per degree, cpd)	VA at Baseline (cycle per degree, cpd)
Light exposure + NGR1 0.025 mg/kg, BID (n = 3)	0.437 ± 0.000	0.109 ± 0.036
Light exposure + NGR1 0.25 mg/kg, BID (n = 3)	0.437 ± 0.000	0.273 ± 0.073

Supplementary table 1. The effective oral doses of NGR1 were evaluated in a pilot study using VA analysis. Mice were administrated NGR1 at 0.025 mg/kg twice daily (BID) and 0.25 mg/kg, BID (n = 3).

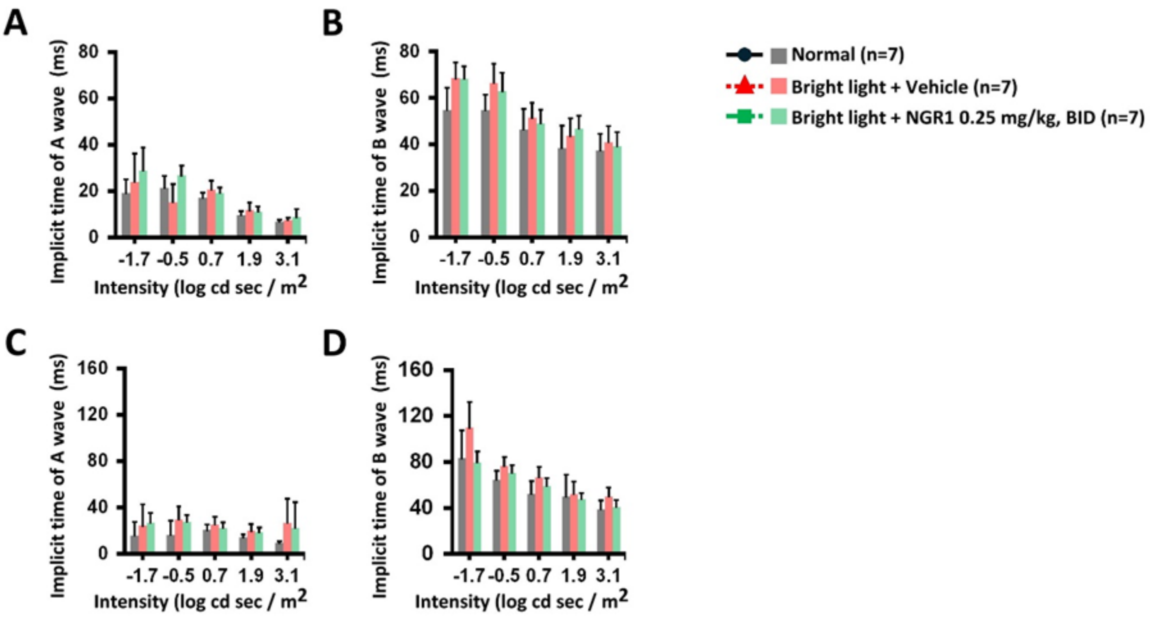

Supplementary Figure 1. (A, B) Average implicit times of a and b waves elicited by green light stimulation under dark-adapted conditions. (C, D) Average implicit times of a and b waves elicited by UV light stimulation under dark-adapted conditions.
